# Deep Multiple Instance Learning Model to Predict Outcome of Pancreatic Cancer Following Surgery

**DOI:** 10.3390/biomedicines12122754

**Published:** 2024-12-02

**Authors:** Caroline Truntzer, Dina Ouahbi, Titouan Huppé, David Rageot, Alis Ilie, Chloe Molimard, Françoise Beltjens, Anthony Bergeron, Angelique Vienot, Christophe Borg, Franck Monnien, Frédéric Bibeau, Valentin Derangère, François Ghiringhelli

**Affiliations:** 1 Cancer Biology Transfer Platform, Georges-François Leclerc Cancer Centre—Unicancer, F-21000 Dijon, France; 2 INSERM, LNC-UMR1231 Research Center, F-21000 Dijon, France; 3 Department of Pathology, CHU Besançon, F-25000 Besançon, France; 4 Department of Pathology, Georges-François Leclerc Cancer Centre—Unicancer, F-21000 Dijon, France; 5 Department of Medical Oncology, CHU Besançon, F-25000 Besançon, France; 6 Department of Medical Oncology, Centre Georges-François Leclerc, F-21000 Dijon, France

**Keywords:** biomarker, pancreatic cancer, deep learning, prognostic

## Abstract

**Background/Objectives**: Pancreatic ductal adenocarcinoma (PDAC) is a cancer with very poor prognosis despite early surgical management. To date, only clinical variables are used to predict outcome for decision-making about adjuvant therapy. We sought to generate a deep learning approach based on hematoxylin and eosin (H&E) or hematoxylin, eosin and saffron (HES) whole slides to predict patients’ outcome, compare these new entities with known molecular subtypes and question their biological significance; **Methods**: We used as a training set a retrospective private cohort of 206 patients treated by surgery for PDAC cancer and a validation cohort of 166 non-metastatic patients from The Cancer Genome Atlas (TCGA) PDAC project. We estimated a multi-instance learning survival model to predict relapse in the training set and evaluated its performance in the validation set. RNAseq and exome data from the TCGA PDAC database were used to describe the transcriptomic and genomic features associated with deep learning classification; **Results**: Based on the estimation of an attention-based multi-instance learning survival model, we identified two groups of patients with a distinct prognosis. There was a significant difference in progression-free survival (PFS) between these two groups in the training set (hazard ratio HR = 0.72 [0.54;0.96]; *p* = 0.03) and in the validation set (HR = 0.63 [0.42;0.94]; *p* = 0.01). Transcriptomic and genomic features revealed that the poor prognosis group was associated with a squamous phenotype. **Conclusions**: Our study demonstrates that deep learning could be used to predict PDAC prognosis and offer assistance in better choosing adjuvant treatment.

## 1. Introduction

Pancreatic ductal adenocarcinoma (PDAC) is the fifth most common digestive cancer in terms of incidence but it is one of the most lethal cancers, with an aggressive behavior and being difficult to diagnose early [1,2,3]. The prognosis is very poor, with a 5-year overall survival rate in PDAC cancer of approximately 10%. Moreover, more than half of the cases are diagnosed at an advanced stage and are not candidates for surgery [4]. It is mostly a disease of older adults, with more than 60% of cases diagnosed in patients over 65 years old, with a median age at diagnosis of 71 years [5]. The incidence of pancreatic cancer is increasing, reflecting the aging population, with an expected 20% increase in adults over 65 years by 2030. Pancreatic cancer is also projected to surpass colorectal cancer in western countries [5].

The current standard of care for treating localized PDAC is based on surgery followed by adjuvant therapy. Surgery is the only curative treatment for PDAC. Only 20% of patients are candidates for tumor resection, while the vast majority of patients are diagnosed with locally advanced or metastatic disease and should therefore be considered for palliative treatment strategies [6,7]. Surgery alone, however, does not cure a majority of patients; median survival is around 10 months and early tumor relapse is observed in most patients. Only 10% of patients are completely cured by surgery [3,8]. Adjuvant chemotherapy has thus been developed during the last decades. Gemcitabine adjuvant therapy was the standard of care for more than, 20 years and this treatment led to major improvements in survival with around 30% of patients relapse-free after 2 years [9,10,11]. More recently, adjuvant chemotherapy with modified folinic acid, fluorouracil, irinotecan and oxaliplatin (mFOLFIRINOX) has strongly improved outcomes, achieving median overall survival of 54.4 months in the mFOLFIRINOX group and about half of the patients being relapse-free after 2 years [12].

Prognostic markers aimed at predicting PDAC recurrence are still an unmet need. Histological evaluation and clinical staging remain the gold standard in the determination of the characteristics of tumor prognostic factors and adjuvant therapy. Emerging prognosis markers are in development, based on the histology-like analysis of fibroblastic tissue whose characteristics are linked to outcome [13] or immune parameters [14]. Similarly, transcriptomic classification by Bailey [15] defined four subtypes (squamous, pancreatic progenitor, immunogenic and aberrantly differentiated endocrine (ADEX)), with the “squamous” subtype having the worst outcome. More recently a transcriptome-based machine learning algorithm was developed to predict response to gemcitabine or FOLFIRINOX [16].

Recent advances in deep learning for computational pathology have enabled the use of whole-slide images (WSIs) to perform cancer diagnosis, analysis of mutational characteristics or determination of prognostic factors in various pathologies. Such algorithms require human expertise, and the detection of novel prognostic morphological features is limited because of the need for human annotations [17]. Histopathological images represent millions of pixels divided into smaller patches, commonly used as input for deep learning models. Most strategies use weakly supervised learning, which relies on slide-level clinical annotation. Weakly supervised learning refers to the fact that there is no a priori knowledge of which patches within the slide are associated with the label of interest. In the context of survival models, a single survival label corresponds to multiple image patches, with one or more patches contributing towards the survival outcome. Thus, one purpose of the model is to discern which patches are relevant to the prediction task. Information from multiple patches in a WSI must then be aggregated to predict one class per slide. The multiple instance learning (MIL) paradigm is one way to deal with this task. In MIL, each patch is represented as an instance in a bag. Since WSIs have more than one patch, the bag contains multiple instances, hence the name “multiple” instance learning. During training, only global (slide-level) image labels are required for supervision. An aggregation mechanism is then used to summarize all the information in instances to make a final prediction.

In this study, we used a survival prediction model based on the attention-guided deep MIL proposed by Yao et al. [18] to determine the prognosis of PDAC treated by surgery; we then characterized patients according to their prognostic groups using transcriptomic and genomic features.

## 2. Materials and Methods

### 2.1. Patients

Two retrospective datasets were used in this study, namely, one training cohort (hereafter called the “Besançon cohort”), and one validation cohort (hereafter “TCGA dataset”).

The Besançon training cohort comprised 206 patients with histologically confirmed PDAC, who underwent complete surgical resection at the University Hospital of Besançon, France, between January 1998 and December 2018 [19]. HES diagnostic whole-slide images (WSIs) and clinical information were available for all of the patients. The database was registered with the National French Commission for bioinformatics data and patient liberty (CNIL) under the number 1906173 v 0. The study methodologies conformed to the standards set by the Declaration of Helsinki. Written informed consent for the use of medical data for research purposes was provided by all patients with cancer at the time of their first visit to the Department of Medical Oncology. Samples were provided by the regional tumor bank of Franche-Comté (University Hospital of Besançon, France; registration number BB-0033-00024). The project was approved by the scientific board of the biobank (#F1860-PAC-MA).

The TCGA dataset comprises 191 H&E diagnostic WSIs from 166 patients, with corresponding molecular (Whole Exome Sequencing (WES) and RNA sequencing) and clinical data. WSIs at X20 magnification, RNAseq raw counts and clinical data, including Bailey classifications, were collected from TCGA via the NIH Genomic Data Commons Data Portal (https://portal.gdc.cancer.gov/, accessed on 5 July 2023). Data were generated by the TCGA Research Network (https://www.cancer.gov/tcga, accessed on 5 July 2023). The inclusion criteria were previously described (https://www.ncbi.nlm.nih.gov/projects/gap/cgi-bin/study.cgi?study_id=phs000178.v11.p8, accessed on 5 July 2023).

The WES biomarkers were obtained from Knijnenburg et al. [20]. WES data were available for 132 patients and RNAseq data for 160 patients.

### 2.2. HES Staining and Numerization

For the Besançon cohort, HES-stained slides from Formalin-Fixed Paraffin-Embedded (FFPE) specimens were digitized with a Nanozoomer HT2.0 (Hamamatsu Photonics, Hamamatsu City, Japan) at 20× magnification to generate WSI files in ndpi format. We partitioned the WSIs into non-overlapping 402 × 402 pixel tiles at 0.5 mm/pixel resolution using QuPath v.0.2.3.

### 2.3. Tile Pre-Processing

Tiles were removed if they contained more than 2/3 of white background. The color channel values were normalized by Macenko [21] normalization to neutralize color differences between slides by bringing them into a common, normalized space. The Macenko method is based on a transformation of the original image into an optical density (OD) space. The primary stained vectors are then estimated using Singular Value Decomposition (SVD), and the image is separated into its stain components. The last step is to normalize these components to a reference distribution and reconstruct the normalized image.

To estimate the deep learning model, we used the Besançon cohort and randomly divided it into training, validation and test sets, as recommended, using, respectively, 60%, 20% and 20% of the tiles. Tiles associated with a given slide were not separated but were associated with one of these three sets to prevent the overlap of slides between sets.

### 2.4. Statistical Analysis

#### 2.4.1. Software

R v4.2.2 was used for statistical analysis. Figures were performed using GraphPad 9.4.1. The deep learning model was implemented and trained using TensorFlow 2.1.0 and Python 3.5. Calculations were performed using HPC resources from DNUM CCUB (Centre de Calcul de l’Université de Bourgogne).

#### 2.4.2. Deep Learning Survival Model

We used a survival prediction model using attention-guided deep MIL, as proposed by Yao et al. [18]. Briefly, the following steps were taken. 1—clustering of tiles from WSIs using k-means approach based on the use of the VGG16 neural network [22] previously trained on ImageNet. These clusters correspond to different phenotypes providing morphology-specific representation. 2—extraction of cluster (phenotype)-level information through a Siamese MIL-based network from tile-level features. The use of this network allows the individual phenotypes provided by the clusters to be taken into account. 3—use of attention mechanism to aggregate these phenotypes features into patient-level information with a trainable weighted average where weights are optimized by neural networks using the negative log partial likelihood of a Cox model as the loss function. The architecture of DeepAttnMISL is presented in the original paper describing the model [18]. The output is risk scores, corresponding to the probability of the survival event occurring. These scores then allow the discrimination of patients into low- or high-risk groups.

#### 2.4.3. Survival Analysis

For survival analysis, the prognostic value of the different variables was tested using univariate or multivariate Cox regression models for progression-free survival (PFS) or overall survival (OS). Univariate models were used to test the association between single variables and the outcome, while multivariate models were used to test the association of multiple variables as predictors. PFS was defined as time to the first relapse or death from any cause. Survival probabilities were estimated using the Kaplan–Meier method, and survival curves were compared using the log-rank test.

Subgroup analysis was conducted on the pooled cohorts to evaluate the effect of adjuvant therapy.

Risk scores were dichotomized using the methodology of Hothorn et al. via the maxstat R library [23]. Using a maximally selected log-rank statistic, this method provides a statically proven optimal cut-off point that corresponds to the most significant relation with the outcome. The resulting dichotomized score yields a variable with two modalities, low or high, according to whether the risk score is above or below the cut-off value.

The flowchart depicted in Supplementary Figure S1 summarizes the different steps of our study.

#### 2.4.4. TCGA RNAseq Analysis

Raw counts already computed with STAR software [24] were downloaded through TCGAbiolinks [25] R library. Gene-level counts were created with the DESeq2 library [26]. Low-count genes were pre-filtered by removing genes with fewer than 5 reads. Genes differentially expressed were then selected using the same R package. Gene set enrichment analysis (GSEA) [27] was performed on resulting differential genes using Hallmarks of cancer gene sets from MSigDB (https://www.gsea-msigdb.org/gsea/msigdb, accessed on 20 February 2024) and the fgsea R package [28].

#### 2.4.5. TCGA Exome Analysis

Exome-derived biomarkers were taken from Knijnenburg et al. [20]. More precisely, we investigated mutational signatures derived by the PanCancer Signature group [29], mutational load, ploidy, aneuploidy score (total number of arm-level amplifications and deletions), Homologous Repair Deficiency (HRD) score, two Loss of Heterozygosity (LOH) scores (total number of segments with LOH events and fraction of genome containing LOH events) and two Copy Number Alteration (CAN) scores (number of segments and fraction of genome altered). The aneuploidy score corresponds to the total number of arm-level amplifications and deletions. Precisions of how these biomarkers were estimated can be found in Knijnenburg et al.

## 3. Results

### 3.1. Dataset Description

To test the performance of the deep learning model for the prediction of outcome in PDAC, we used two large datasets with high-resolution WSIs. The Besançon cohort, used as the training set, comprised 206 patients and contained 7,147,508 tiles, ranging from 11,052 to 56,446 tiles per slide (median = 34,890 tiles). The validation cohort came from the TCGA database and contained 191 slides from 166 patients, with 3,671,750 tiles, ranging from 1128 to 46,304 tiles per slide (median = 18,578 tiles). Patients with neuroendocrine carcinoma (N = 8) were excluded from analysis because this type of tumor was not included in the Besançon cohort. In addition, these tumors have different treatment and prognosis than adenocarcinoma and could therefore bias the analysis. WSIs from the training cohort used HES staining, while TCGA used H&E staining only. All the available clinical characteristics of the patients are summarized in Table 1.

The training cohort includes 101 (50%) male patients while TCGA includes 96 (55%) male patients. The mean age was 67 years old for the training dataset and 66 years for the TCGA cohort. In the training cohort, 146 (76%) patients received adjuvant therapy while 66 (56%) patients for whom information was available received adjuvant therapy in TCGA. Median OS was 21.9 months (95% CI 18.4; 25.7) and 19.2 (95% CI 16.5; 21.5) months for the Besançon and TCGA cohorts, respectively. Median PFS was 10.10 months (95% CI 9.4; 11.5 months) for the Besançon cohort and 14.7 months (95% CI 12.6; 17.0 months) for the TCGA cohort.

### 3.2. Estimation of the Attention-Guided Multiple Instance Learning Model

To estimate a robust deep learning model to learn survival patterns, the training cohort was separated into internal training, validation and test sets, with a 60%–20%–20% distribution.

As explained in Section 2, image patches were first clustered for each patient, leading to phenotype clusters with patches sharing common appearance. For the k-means step, k was set to 10 clusters. Examples of phenotype pattern visualization after clustering are depicted on three WSIs from two distinct patients in Supplementary Figure S2.

To learn patient-level information from phenotype clusters, a Multiple Instance Fully Convolutional Network (MI-FCN) running inside the deep learning architecture was used, with weights being shared among them, as in the Siamese architecture [18]. The output of the model is the hazard survival risk, corresponding to the probability of the progression-free survival event occurring. The estimated risk scores were then used to classify patients into low- or high-risk groups, based on the best cut-off strategy proposed by Hothorn et al. [23].

**Figure 1:**
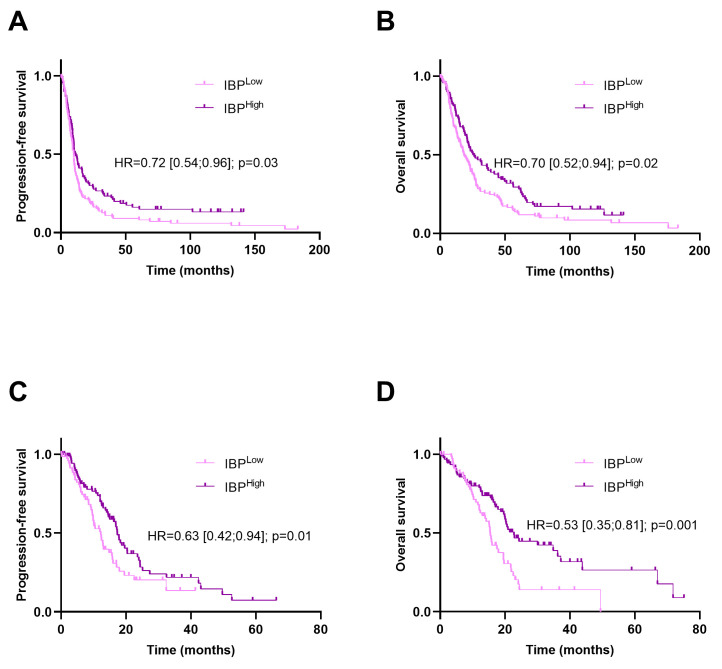
Kaplan–Meier curves for progression-free and overall survival with patients stratified according to the image-based prognostic (IBP) score in the Besançon cohort (**A**,**B**) and the TCGA cohort (**C**,**D**).

We tested various models using 5-fold cross-validation and 10 to 80 epochs. Using the Besançon cohort, the model yielding the best accuracy in the internal test set was retained and showed an association between a high score and a good outcome. A high score was associated with an HR of 0.72 [0.54;0.96]; *p* = 0.03 for PFS and 0.70 [0.52;0.94]; and *p* = 0.02 for OS in the training cohort (Figure 1A,B). In the TCGA validation set, using the same threshold, a high score was associated with an HR of 0.62 [0.42;0.94]; *p* = 0.01 for PFS and 0.53 [0.38;0.81]; and *p* = 0.001 for OS (Figure 1C,D).

**Figure 2:**
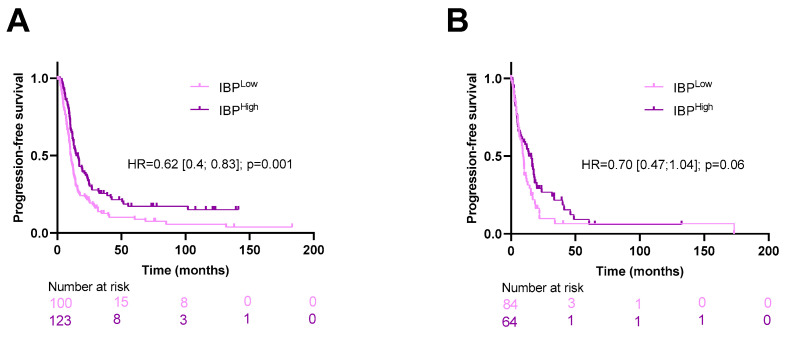
Kaplan–Meier curves for progression-free survival with patients stratified according to the image-based prognostic (IBP) score in the pooled cohort for patients treated (**A**) or untreated with adjuvant therapy (**B**).

Upon subgroup analysis in our study, our model remained significant in patients treated with adjuvant therapy (HR = 0.62 [0.4;0.83]; *p* = 0.001), and close to significance in untreated patients (HR = 0.70 [0.47;1.04]; *p* = 0.06); this analysis was conducted on the pooled cohorts to increase statistical power (Figure 2).

### 3.3. Generation of a Composite Clinical and Image-Based Prognostic Score

We next explored the capacity to generate a prognostic composite model associating an image-based prognostic (IBP) score and clinical features in the training set. To increase the power of the analysis, we pooled the two cohorts. We built a multivariate model using each clinical variable significantly associated with PFS by univariate Cox proportional hazards analysis, namely, resection status, nodal status and histological grade; only nodal status and histological grade remained significant in the multivariate model (Table 2). For OS, the same three variables were significant in the univariate and multivariate models (Supplementary Table S1).

We then generated a combined model with image-based and clinical prognostic variables. We observed that the combined score had a strong prognostic value for PFS and OS (Table 3 and Supplementary Table S2).

AUC comparisons showed that the combined score had added value over the clinical score (Likelihood Ratio test *p* = 0.005) and image-based score (Likelihood Ratio test: *p* < 1.10^−3^).

**Figure 3:**
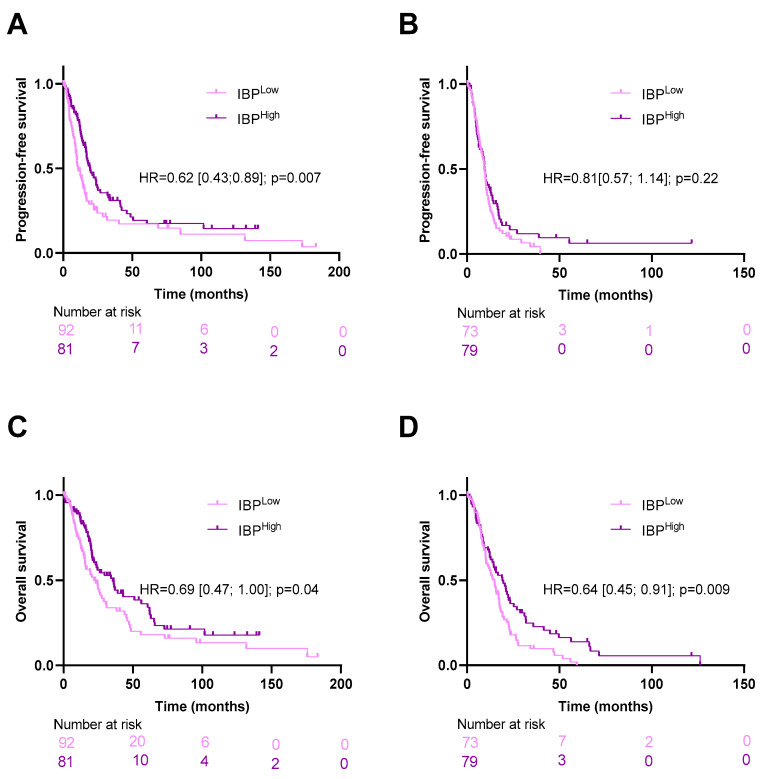
Kaplan–Meier curves for progression-free and overall survival with patients stratified according to the image-based prognostic (IBP) score in the pooled cohort, respectively, for patients with low (**A**,**C**) and high (**B**,**D**) clinical risk.

However, when we split the patients into two groups of clinical risk of relapse, we observed that the deep learning model yielded additional prognostic information only in the low clinical risk score group for PFS and in both groups for OS (Figure 3).

### 3.4. Interpretability of the Deep Learning Image Model

**Figure 4:**
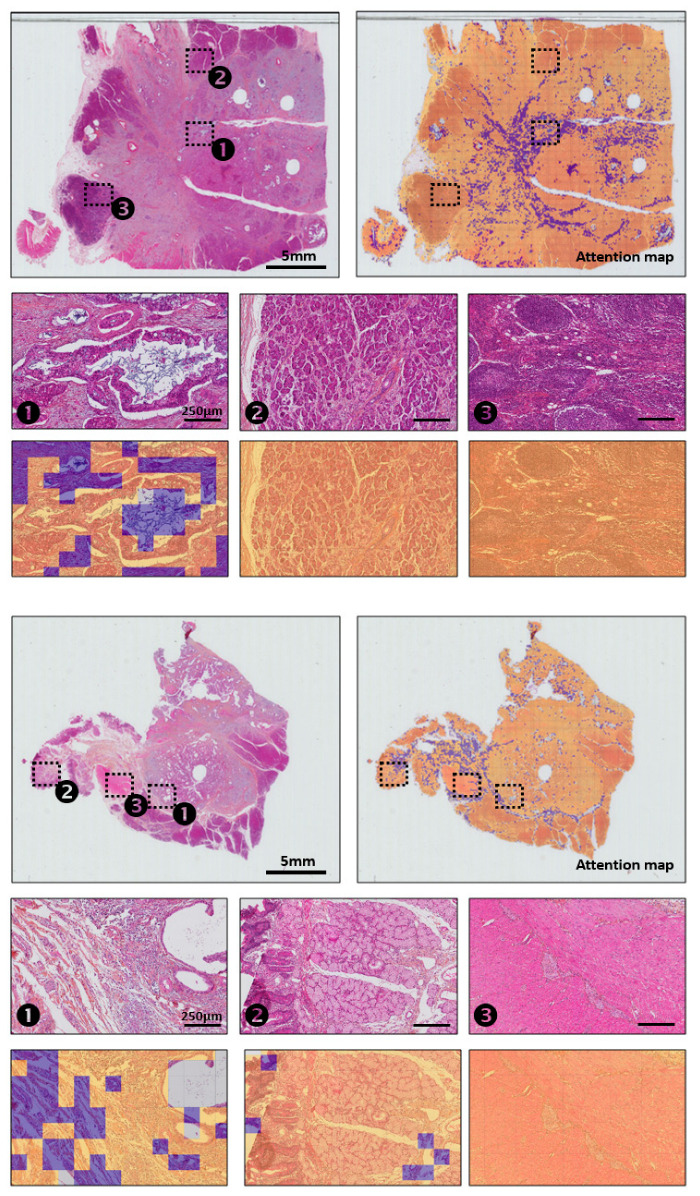
HES images and corresponding attention maps reflecting attention weight patterns for 2 patients. Blue color corresponds to the highest-level attention tiles. Numbers 1 to 3 refer to 3 zones on each slide that were highlighted and for which a zoom was provided.

The attention mechanism in DeepAttnMISL makes it possible to detect important phenotypes associated with patients’ clinical outcomes. To determine the histological pattern associated with the attention mechanism, we collected and examined the attention weights as well as their corresponding patch images for several representative slides of patients split into low or high IBP groups (Figure 4).

We asked two pathologists to evaluate low- and high-attention weight regions to add an interpretative layer to our results. Interpretations were hard to establish. On the slides depicted in Figure 4, the peritumoral areas (1) (upper and lower slides) showed high attention mainly on the fibrosis region and less on the epithelial cells. Some attention, particularly on the upper slide, was focused on the mucin content. Little or no attention was focused on the healthy regions such as the exocrine pancreatic glands, immune patches ((2) and (3) on upper slide), duodenum mucosa and muscularis propria ((2) and (3) on lower slide).

For some slides, low attention was given to normal tissue and background, and high attention was given to the tumor area; these observations were not valid for all slides. It was hard to find homogeneous patterns of relapse. Moreover, some patterns that were identified were not intuitive to pathologists. These observations are consistent with the difficulty pathologists encounter in identifying patients with a low or high risk of relapse.

### 3.5. Description of Molecular Characteristics

**Figure 5:**
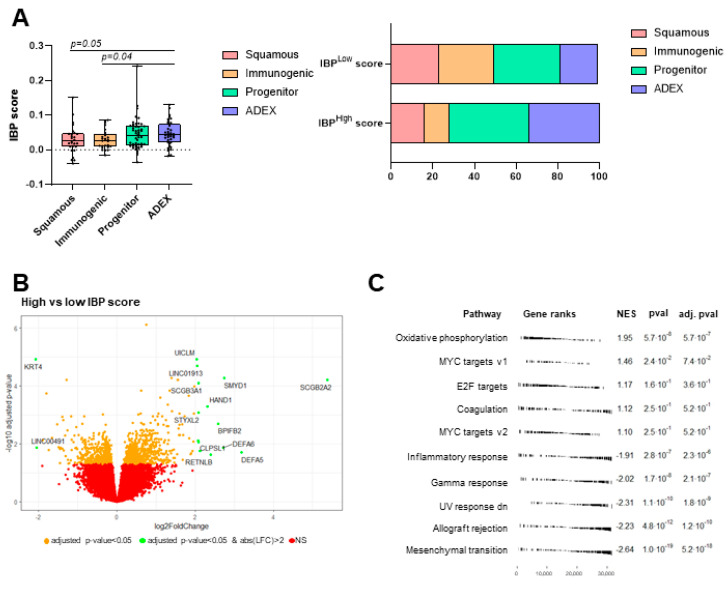
(**A**) Left: Boxplot displaying image-based prognostic score depending on squamous molecular subtype. Each point corresponds to the IBP score of one patient. Right: Barplot displaying the repartition of molecular subtypes for patients with low and high image-based prognostic score. (**B**) Volcano plot displaying differentially expressed genes, given IBP score. The vertical axis (y-axis) corresponds to the mean expression value of log 10 of adjusted *p* value using Benjamini–Hochberg FDR correction, and the horizontal axis (x-axis) displays the log 2 fold change value. Green dots on the right (or left, respectively) are genes significantly upregulated (or downregulated) in patients with high IBP score. (**C**) Pathway classification from RNA-seq results using gene set enrichment analysis based on the hallmark of cancer database comparing patients with high vs low IBP scores. NES: normalized enrichment score.

To go further in the analysis of the biological differences between the two image-based prognostic groups, we used whole exome and RNAseq from the TCGA dataset. Bailey transcriptomic classification was obtained for 139 patients. Using this classification, patients were split into four categories: squamous for 27 patients, immunogenic for 26 patients, progenitor for 49 patients and ADEX for 37 patients. In this dataset, the squamous molecular subtype was associated with poor PFS. The squamous and immunogenic subsets presented a significantly lower IBP score compared to ADEX and progenitor type tumors. The low IBP score population was significantly enriched in squamous and immunogenic tumors compared to ADEX tumors (Figure 5A). However, the IBP score is not a perfect reflection of molecular classification.

Using differential expression analysis, we compared gene expression between low and high IBP scores (Figure 5B). With a cut-off of a log fold change of 2 and adjusted *p*-value < 0.05, only high KRT4 expression and LINC00491 were associated with a low IBP score. In contrast, 12 genes were significantly more highly expressed in patients with a high IBP score. Using EnrichR software (v1) and the Reactome 2022 database, these 12 genes were enriched in the Alfa-defensins pathway (*p*-value < 1.10^−3^). Using gene set enrichment analysis and Hallmarks of cancer, we observed that oxidative-phosphorylation, E2F and MYC targets, DNA repair and coagulation pathways were upregulated in high IBP score tumors, while inflammatory response, gamma response, UV response, allograft rejection and mesenchymal transition were upregulated in low IBP score tumors (Figure 5C).

Using genomic TCGA analysis, we observed that KRAS and TP53 are the most frequently mutated genes (Supplementary Figure S3). No differences in single nucleotide variants between the two IBP groups were observed. Using mutational signature analysis of the WES data through SBS96 classification, we observed that only signatures 48 and 91 were highly expressed in the low IBP group. However, the SBS91 signature was expressed only in three patients (all with low IBP score), and SBS48 was expressed only in seven patients, leading us to consider these observations with caution.

## 4. Discussion

This work presents an automated weakly supervised deep learning method to predict PFS and OS of localized PDAC treated by surgery based on the analysis of a single whole H&E or HES slide. Our digital marker allowed the automatic prediction of PFS and OS of PDAC with an HR around 0.7 when comparing patients with low and high digital score. We validated our results in an independent public dataset, thus strengthening our conclusions. Using AUC, we could demonstrate that the deep learning model improved prediction capacity when compared to clinical variables alone, thus suggesting its interest in clinical practice. Upon subgroup analysis, the additive predictive information of the deep learning model seemed more powerful in patients with good clinical-based prognostic and in patients who received adjuvant therapy, thus suggesting that it could be a clinically relevant tool to help with decisions regarding adjuvant therapy in patients with small tumors.

When trying to characterize IBP-based prognostic groups, we showed that high KRT4 and LINC00491 expression was associated with a low IBP score. KRT4 is a marker of squamous differentiation. This squamous differentiation is a well-known biomarker of poor prognosis in pancreatic cancer [30]. The gene encodes for Keratin 4, a type II cytokeratin consisting of basic or neutral proteins that are arranged in pairs of heterotypic keratin chains, co-expressed during the differentiation of simple and stratified epithelial tissues. In normal tissues, this cytokeratin is specifically expressed in esophageal epithelia, and it is expressed in most squamous tumors. This could be linked to the observation that the population with a low IBP score was significantly enriched in squamous subtypes, associated with poor PFS. The mesenchymal transition pathway was also associated with low IBP score. Among the genes involved in the EMT signature, we observed that 12 genes (VCAN, COL11A1, ITGB5, TIMP3, INHBA, LRP1, MATN3, NOTCH2, CDH11, IGFBP3, COL8A2, THBS2) were significantly (adjusted *p*-values below 0.05) associated with low IBP. LINC00491 is a long non-coding RNA that was previously associated with aggressive features of pancreatic cancer with high proliferation and invasive capacities [31]. In contrast, a high IBP score is associated with high expression of alfa defensin-related genes. Alfa defensin-related genes were previously described as being highly expressed in various cancers and DEFA5 was described as a potent suppressor of cancer growth [32].

Deep learning strategies are emerging in pancreatic cancer. A recent review by Patel et al. [33] gave an overview of the applications of deep learning (DL) in the diagnosis, management and monitoring of patients diagnosed with pancreatic cancer. Most methods propose models for diagnosis rather than for prognosis. Among others, the authors investigated applications in treatment response. They cited a study from Watson et al. [34] conducted on 81 patients for whom preoperative cross-sectional imaging was available; the authors proposed a composite model combining biomarkers derived from a preoperative imaging model (CNN based on LeNet architecture) and a CA 19-9 model, achieving an AUC of 0.79. To our knowledge, they did not provide any interpretation layer. A number of studies used radiomic to determine pancreatic cancer prognostic factors [35,36,37]. A deep learning-based computed tomograpy imaging-derived score enabled the prediction of OS for patients with resectable PDAC. Similarly, a multiomics approach could also be analyzed using a deep learning model to predict outcome. Using autoencoder integrated multiomics of 146 patients with PDAC enabled the identification of two PDAC subtypes with distinct survival outcomes (median survival 10.1 and 22.7 months) [38]. Using pathological slides, some models are available to improve tumor detection on histological slides or to improve prediction [39]. In contrast, very few deep learning models were trained to predict prognostic factors of PDAC. A recent paper showed interest in using machine learning to predict survival in pancreatic neuroendocrine carcinoma [40]. In the field of PDAC, Pacpaint software (v1.0.0) was developed as a multi-level artificial intelligence-based tool using deep learning models to determine PDAC molecular subtypes, thus enabling intra-tumor heterogeneity to be deciphered. Pacpaint highlights the presence of aggressive basal contingent and thus helps to detect patients with a poor outcome [41]. Accordingly, in our study, we observed that a low image-based deep learning score, associated with poor prognosis, was also associated with a higher incidence of the squamous phenotype upon transcriptomic analysis but did not strictly reproduce the molecular classification of PDAC, thus suggesting that our model may detect squamous features and probably some parameters other than molecular classification. However, we could not define clear pathological structures associated with outcome. In fact, the interpretation of low- and high-attention weight regions derived from the model by pathologists was hard to establish. We believe that this is consistent with the fact that currently, it is also difficult for pathologists to find patterns associated with the risk of relapse. Our model may highlight particularities that are not evident to the naked eye.

Only one recent report using pan-cancer data underlined the capacity to generate a deep learning-based multimodal fusion algorithm that uses both H&E WSIs and molecular profile features (mutation status, copy-number variation, RNA sequencing expression) to measure and explain the relative risk of cancer death. The protocol used an attention-based multiple instance learning model (AMIL) with WSIs in 14 types of cancers. In this study, the AMIL could not predict PDAC survival with H&E WSIs alone, whereas the association of H&E WSIs and molecular data was powerful in predicting survival [42]. In our study, our model differs in the first clustering step, which allows the definition of different patterns for each patient; these phenotype clusters are considered as the instance of the bag instead of individual patches. Such a difference may explain the better discrimination power of our deep learning tool.

Using the machine learning approach in a small dataset, Nimgaonkar et al. [43] showed that an image analysis pipeline involving nuclei segmentation, the extraction of 816 features describing nuclear morphology, and feature selection using least absolute shrinkage and selection operator (LASSO) regression could be used to predict outcome in patients treated with gemcitabine in an adjuvant setting. Upon subgroup analysis in our study, our model remains significant in patients treated with adjuvant therapy, suggesting that deep learning using H&E WSIs could be used to improve prediction of the efficacy of adjuvant therapy.

However, there were some limitations to our study. First, this study only included data from retrospective and heterogeneous cohorts. The adjuvant treatment mainly consisted of gemcitabine, which is not the current standard of care; FOLFIRINOX is now recommended for these patients. Analysis of the predictive power of our deep learning model in a prospective series of patients treated with FOLFIRINOX would provide more reliable evidence of the actual predictive performance of the model.

## 5. Conclusions

In summary, we presented an automated histological-based deep learning model to predict outcome in localized pancreatic cancer. This model added predictive information to clinical data and confirmed the feasibility of using computational histopathology through deep learning to improve pancreatic cancer management.

## Figures and Tables

**Table 1:** Summary of clinical characteristics of patients from the Besançon (N = 206) and TCGA (N = 166) cohorts.

Variables	Besançon Cohort, N = 206 ^1^	TCGA Cohort, N = 166 ^1^	*p*-Value ^2^
Sex		data	0.43
F	103 (50%)	74 (45%)	
M	101 (50%)	92 (55%)	
Unknown	1	-	
Age	67 (40, 86)	66 (36; 89)	
Unknown	1	-	
Neoadjuvant treatment			-
No	194 (95%)	165 (99%)	0.03
Yes	10 (5%)	1 (1%)	
Unknown	1	-	
Adjuvant treatment			<1.10^−3^
No	46 (24%)	51 (44%)	
Yes	146 (76%)	66 (56%)	
Unknown	13	49	
Resection			0.003
0	161 (80%)	99 (65%)	
1	37 (18%)	49 (32%)	
2	2 (2%)	5 (3%)	
Unknown	5	13	
Histological grade			0.003
1	42 (21%)	23 (14%)	
2	125 (61%)	90 (55%)	
3	32 (16%)	50 (30.5%)	
4	5 (2%)	1 (0.5)%	
Unknown	1	2	
Tumor status			<1.10^−3^
1	24 (14%)	6 (4%)	
2	110 (64%)	23 (11%)	
3	39 (23%)	137 (83%)	
4	0 (0%)	4 (2%)	
Node status			<1.10^−3^
0	49 (24%)	45 (27.5%)	
1	83 (41%)	115 (72.5%)	
2	72 (35%)	0	
Unknown	1	2	

^1^ Median (min, max); n (%); ^2^ Wilcoxon rank sum test; Pearson’s chi-squared test; Fisher’s exact test.

**Table 2:** Univariate and multivariate Cox models for clinical variables and progression-free survival in the Besançon and TCGA pooled cohorts.

	Univariate				Multivariate		
Variables	N	HR ^1^	95% CI ^1^	*p*-Value	HR ^1^	95% CI ^1^	*p*-Value
Histological grade	370						
1		—	—		—	—	
2		1.16	0.85, 1.60	0.35	1.37	0.98, 1.91	0.063
3		1.87	1.29, 2.70	<0.001	2.22	1.50, 3.30	<0.001
4		1.33	0.51, 3.43	0.56	1.78	0.61, 5.21	0.29
Age	372	1.01	1.00, 1.02	0.25			
Sex	372						
F		—	—				
M		0.86	0.69, 1.09	0.21			
Tumor size status	343						
1		—	—				
2		1.26	0.80, 1.97	0.32			
3		1.07	0.69, 1.67	0.77			
Node status	370						
0		—	—		—	—	
1		1.34	1.00, 1.79	0.053	1.25	0.92, 1.70	0.15
2		2.03	1.45, 2.83	<0.001	2.05	1.44, 2.91	<0.001
Resection	356						
0		—	—				
1		1.56	1.20, 2.03	0.001	1.52	1.16, 2.00	0.002
2		3.16	1.48, 6.75	0.003	2.52	1.17, 5.43	0.018
Adj. treatment	371						
0		—	—				
1		0.82	0.64, 1.04	0.10			

^1^ HR = hazard ratio, CI = confidence interval.

**Table 3:** Univariate and multivariate Cox models for variables included in the combined model and progression-free survival in the Besançon and TCGA pooled cohorts.

	Univariate				Multivariate		
Variables	N	HR ^1^	95% CI ^1^	*p*-Value	HR ^1^	95% CI ^1^	*p*-Value
Histological grade	370						
1		—	—		—	—	
2		1.16	0.85, 1.60	0.35	1.37	0.98, 1.91	0.065
3		1.87	1.29, 2.70	<0.001	2.28	1.54, 3.38	<0.001
4		1.33	0.51, 3.43	0.56	1.55	0.53, 4.55	0.42
Resection	356						
0		—	—		—	—	
1		1.56	1.20, 2.03	0.001	1.51	1.15, 1.97	0.003
2		3.16	1.48, 6.75	0.003	2.60	1.21, 5.59	0.015
Node status	370						
0		—	—		—	—	
1		1.34	1.00, 1.79	0.053	1.23	0.91, 1.68	0.18
2		2.03	1.45, 2.83	<0.001	1.95	1.37, 2.77	<0.001
IBP score	372						
Low		—	—		—	—	
High		0.67	0.53, 0.85	<0.001	0.71	0.55, 0.90	0.001

^1^ HR = hazard ratio, CI = confidence interval.

## Data Availability

Data are available on request only, due to ethical reasons.

## Supplementary Materials

The following supporting information can be downloaded at: https://www.mdpi.com/article/10.3390/biomedicines12122754/s1, Figure S1: Flowchart of study; Figure S2: Phenotype pattern visualization after clustering on 2 WSIs coming from 2 distinct patients; Figure S3: Oncoplot of the 10 most frequent somatic mutations identified in (A) all sequenced TCGA samples (N  =  164), (B) IBP^Low^ (N = 67) and (C) IBP^High^ (N = 97) score patients. Percentage frequency of the genes is shown in the barchart to the right of the central plot. Continuous IBP scores are indicated under the central plot. Table S1: Univariate and multivariate Cox models for clinical variables and overall survival in the Besançon and TCGA pooled cohorts; Table S2: Univariate and multivariate Cox models for variables included in the combined model and overall survival in the Besançon and TCGA pooled cohorts.
