# Supplementary material for: Deep Multiple Instance Learning Model to Predict Outcome of Pancreatic Cancer Following Surgery
Source: Biomedicines. 2024 Dec 2;12(12):2754. doi: 10.3390/biomedicines12122754 (PMC11673784; doi:10.3390/biomedicines12122754)
Supplement: Supplementary file 1 [file biomedicines-12-02754-s001.zip › biomedicines-3262417-supplementary.pdf]

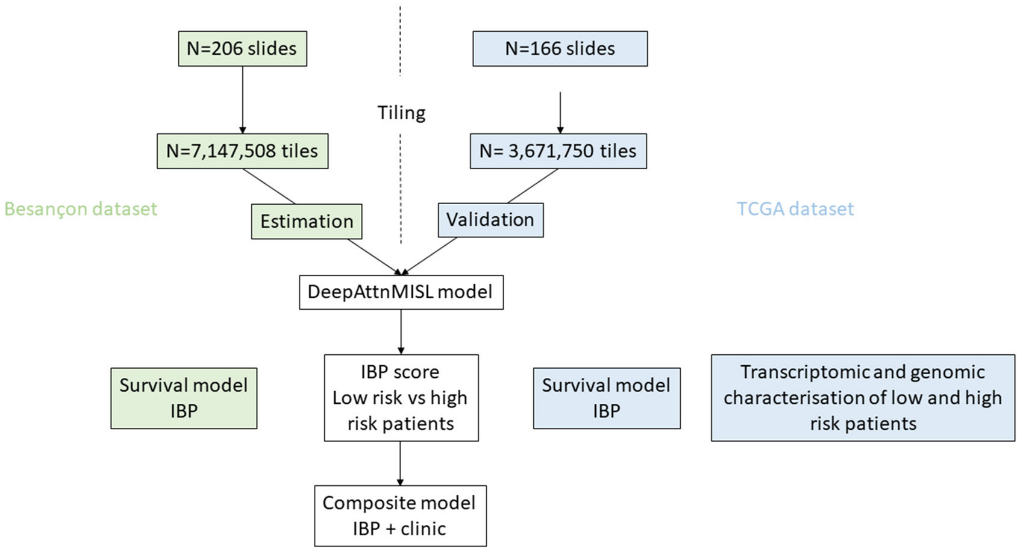

Figure S1. Flowchart of study.

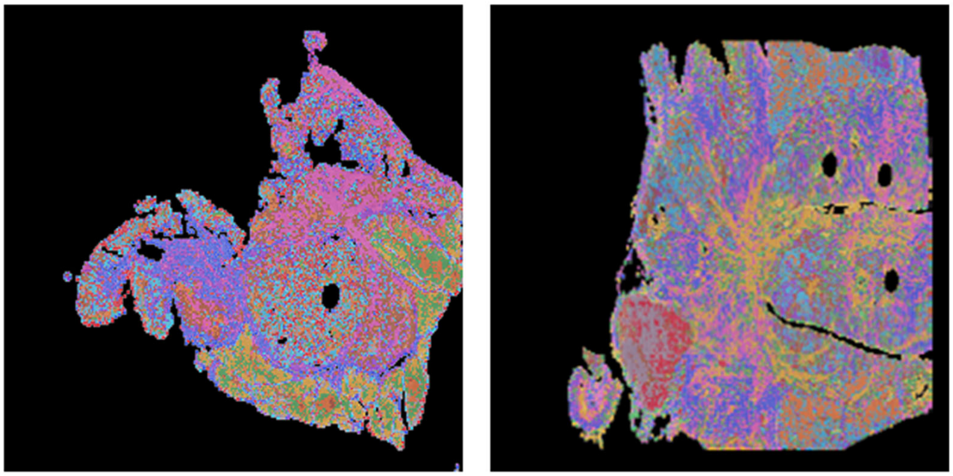

Figure S2. Phenotype pattern visualization after clustering on 2 WSIs coming from 2 distinct patients

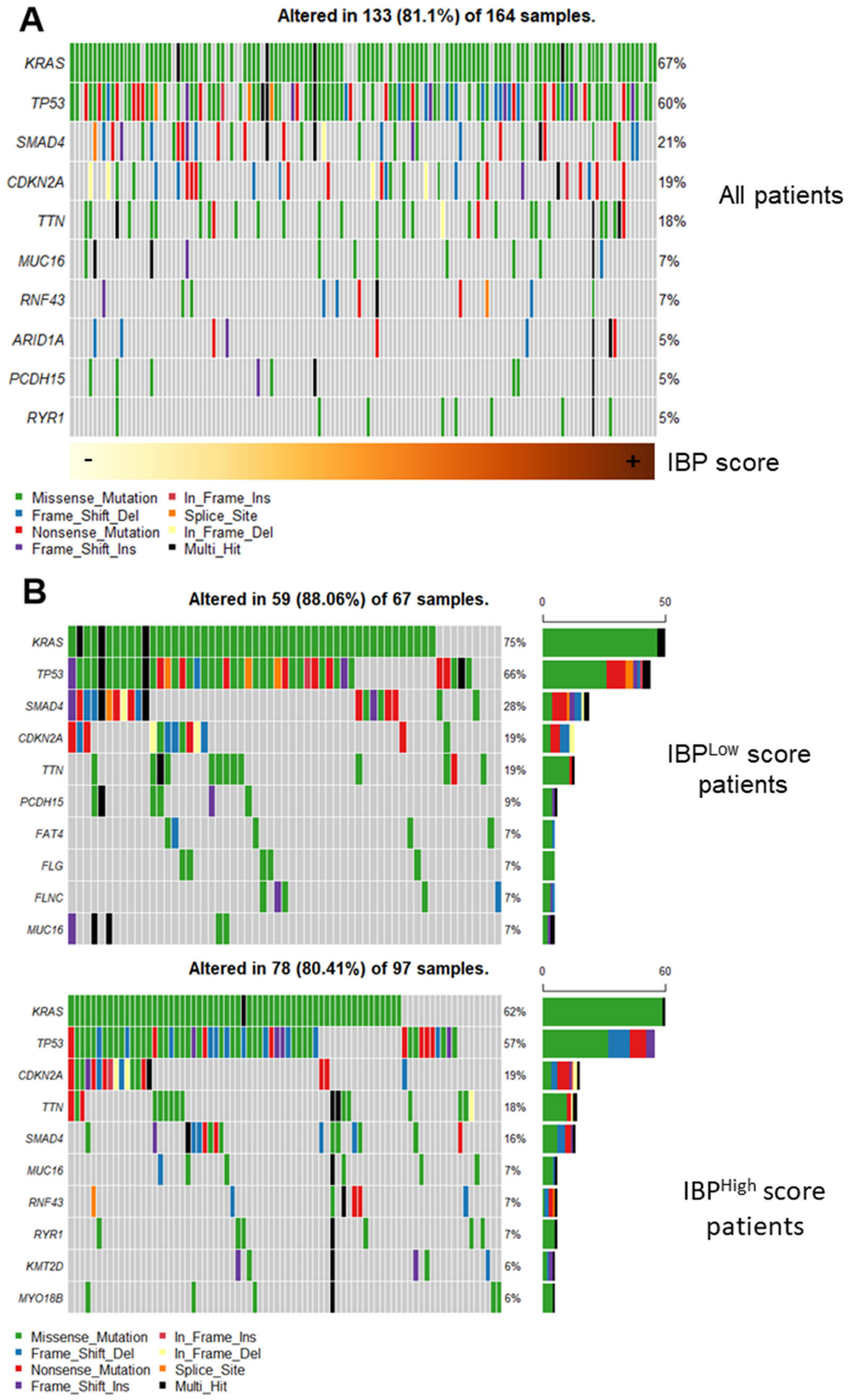

Figure S3. Oncoplot of the 10 most frequent somatic mutations identified in (A) all sequenced TCGA samples (N = 164), (B) IBP^{Low} (N = 67) and (C) IBP^{High} (N = 97) score patients. Percentage frequency of the genes is shown in the barchart to the right of the central plot. Continuous IBP scores are indicated under the central plot

Table S1. Univariate and multivariate Cox models for clinical variables and overall survival in the Besançon and TCGA pooled cohorts

		Univariate			Multivariate		
**Characteristic**	**N**	**HR[1]**	**95% CI[1]**	**p-value**	**HR[1]**	**95% CI[1]**	**p-value**
Histological grade	370						
1		—	—		—	—	
2		1.29	0.92, 1.80	0.14	1.44	1.02, 2.03	0.039
3		2.12	1.44, 3.11	<0.001	2.47	1.65, 3.68	<0.001
4		1.16	0.45, 2.98	0.76	1.47	0.51, 4.22	0.48
Age	372	1.01	1.00, 1.03	0.023			
Sex	372						
F		—	—				
M		0.77	0.61, 0.98	0.036			
Tumor size status	343						
1		—	—				
2		1.47	0.92, 2.34	0.11			
3		1.44	0.90, 2.29	0.13			
Node status	370						
0		—	—		—	—	
1		1.53	1.12, 2.11	0.008	1.51	1.08, 2.11	0.015
2		2.06	1.44, 2.93	<0.001	2.08	1.43, 3.02	<0.001
Resection	356						
0		—	—		—	—	
1		1.49	1.13, 1.96	0.004	1.46	1.11, 1.93	0.007
2		1.62	0.60, 4.38	0.34	1.25	0.46, 3.41	0.66
Adj. treatment	371						

		Univariate			Multivariate		
Characteristic	N	HR[1]	95% CI[1]	p-value	HR[1]	95% CI[1]	p-value
0		—	—				
1		0.59	0.46, 0.76	<0.001			

[1]HR = Hazard Ratio, CI = Confidence Interval

Table S2. Univariate and multivariate Cox models for variables included in the combined model and overall survival in the Besançon and TCGA pooled cohorts

		Univariate			Multivariate		
Characteristic	N	HR[1]	95% CI[1]	p-value	HR[1]	95% CI[1]	p-value
Histological grade	370						
1		—	—		—	—	
2		1.29	0.92, 1.80	0.14	1.45	1.03, 2.05	0.035
3		2.12	1.44, 3.11	<0.001	2.54	1.70, 3.80	<0.001
4		1.16	0.45, 2.98	0.76	1.35	0.47, 3.90	0.58
Resection	356						
0		—	—		—	—	
1		1.49	1.13, 1.96	0.004	1.45	1.10, 1.92	0.008
2		1.62	0.60, 4.38	0.34	1.31	0.48, 3.57	0.59
Node status	370						
0		—	—		—	—	
1		1.53	1.12, 2.11	0.008	1.49	1.07, 2.08	0.020
2		2.06	1.44, 2.93	<0.001	2.01	1.38, 2.91	<0.001
IBP score	372						
Low		—	—		—	—	
High		0.65	0.52, 0.83	<0.001	0.69	0.54, 0.69	0.001

[1]HR = Hazard Ratio, CI = Confidence Interval
